# CORVET-specific subunit levels determine the balance between HOPS/CORVET endosomal tethering complexes

**DOI:** 10.1038/s41598-024-59775-0

**Published:** 2024-05-02

**Authors:** Ármin Sőth, Márton Molnár, Péter Lőrincz, Zsófia Simon-Vecsei, Gábor Juhász

**Affiliations:** 1 Department of Anatomy, Cell and Developmental Biology, Eötvös Loránd University (ELTE), Pázmány Péter sétány 1/C, Budapest, 1117 Hungary; 2 Momentum Vesicle Trafficking Research Group, Hungarian Academy of Sciences-Eötvös Loránd University, Budapest, Hungary; 3 Momentum Lysosomal Degradation Research Group, Institute of Genetics, HUN-REN Biological Research Centre Szeged, Szeged, Hungary

**Keywords:** Membrane fusion, Protein-protein interaction networks

## Abstract

The closely related endolysosomal tethering complexes HOPS and CORVET play pivotal roles in the homo- and heterotypic fusion of early and late endosomes, respectively, and HOPS also mediates the fusion of lysosomes with incoming vesicles including late endosomes and autophagosomes. These heterohexameric complexes share their four core subunits that assemble with additional two, complex-specific subunits. These features and the similar structure of the complexes could allow the formation of hybrid complexes, and the complex specific subunits may compete for binding to the core. Indeed, our biochemical analyses revealed the overlap of binding sites for HOPS-specific VPS41 and CORVET-specific VPS8 on the shared core subunit VPS18. We found that the overexpression of CORVET-specific VPS8 or Tgfbrap1 decreased the amount of core proteins VPS11 and VPS18 that are assembled with HOPS-specific subunits VPS41 or VPS39, indicating reduced amount of assembled HOPS. In line with this, we observed the elevation of both lipidated, autophagosome-associated LC3 protein and the autophagic cargo p62 in these cells, suggesting impaired autophagosome-lysosome fusion. In contrast, overexpression of HOPS-specific VPS39 or VPS41 did not affect the level of assembled CORVET or autophagy. VPS8 or Tgfbrap1 overexpression also induced Cathepsin D accumulation, suggesting that HOPS-dependent biosynthetic delivery of lysosomal hydrolases is perturbed, too. These indicate that CORVET-specific subunit levels fine-tune HOPS assembly and activity in vivo.

## Introduction

Living organisms require coordinated operation of biosynthetic and degradative pathways for maintaining cellular homeostasis. To achieve this, eukaryotic cells sustain various membrane-bound organelles, and proteins and lipids are constantly exchanged between them via vesicle transport. This process depends on multiple fusion and fission events. Vesicular fusion is executed by a conserved machinery consisting of Rab GTPases, their interacting effectors, and SNARE (soluble NSF attachment protein receptor) proteins that are found on both membranes^1^. The maturation of endosomes is regulated by Rab proteins, and their GTP-bound, active form can bind multiple effectors, such as tethering factors. These can bring the donor and acceptor membranes into contact and thus facilitate the homo- and heterotypic fusion of vesicles^2^. The fusion is completed by SNAREs that mediate the mixing of the lipid bilayers^1^.

Endolysosomal tethering complexes, namely CORVET (Class C core endosome vacuole tethering) and HOPS (Homotypic vacuole fusion and protein sorting) were first identified in yeast^3–5^. The homotypic fusion of VPS (Vacuolar protein sorting)21/Rab5-positive early endosomes is mediated by CORVET^6^, whilst HOPS promotes the fusion of Ypt7/Rab7-positive late endosomes as well as autophagosomes with lysosomes^7–9^. In yeast, the complexes consist of four shared, core subunits (VPS16, VPS33, VPS18, VPS11), and two complex-specific subunits: VPS8 and VPS3 for CORVET^5^ and VPS41 and VPS39 for HOPS^33,44^. The specific subunits are responsible for targeting the complexes to specific membranes by binding to membrane-bound (thus GTP loaded) Rab small GTPases (Rab2 and Rab7 in the case of HOPS and Rab5 for CORVET in animal cells)^10–13^ and hence they bring the fusion-primed vesicles closer to each other.

In Drosophila, a tetrameric miniCORVET complex was identified, which lacks VPS11 and VPS3 (the latter one has no homologs in higher eukaryotes)^14^. In mammals, CORVET harbours the VPS39 ortholog Tgfbrap1 instead of VPS3^15,16^, whilst HOPS is conserved in all metazoans.

The existence of shared subunits raises the possibility of interconversion between HOPS and CORVET, and hence the formation of hybrid complexes. Biochemical experiments in yeast identified complexes containing the class C VPS proteins (VPS16, VPS33, VPS18 and VPS11) and one CORVET- and one HOPS-specific subunits: “i-HOPS”: VPS8-VPS39 and “i-CORVET”: VPS41-VPS3^5,17^. In Drosophila, the only possible hybrid complex (VPS8-VPS39) could not be found based on mass spectrometry data for interacting proteins of CORVET-specific VPS8, and our functional and localization analyses suggested that these complexes assemble independent of each other^14,18^.

The shared class C core subunits and overlapping binding sites^17,19^ allows potential competition between the complex specific subunits, which can affect the intracellular ratio of these complexes. In yeast, VPS3 overexpression decreases the level of assembled HOPS and leads to vacuole fragmentation, which resembles to the VPS39 (HOPS) loss of function phenotype. However, overproduction of VPS8 did not have such effect^5^. In contrast, overexpression of Drosophila VPS8, the only miniCORVET-specific subunit, inhibits HOPS-dependent trafficking routes by outcompeting VPS41 from HOPS, while increased amount of HOPS-specific VPS41 does not disturb CORVET functions^18^. Based on an electron microscopy-based structure of the yeast HOPS complex^2^ and detailed characterization of the architecture of mammalian complexes^19^, a similar competition can occur between the specific subunits of the human complexes as well. However, this question has not been investigated yet in mammalian cells.

Hereby we show that overexpression of human CORVET-specific VPS8 or Tgfbrap1 decreased the intracellular level of HOPS, while the gain of their HOPS-specific counterparts, VPS41 and VPS39, respectively, did not affect CORVET. Additionally, high level of VPS8 or Tgfbrap1 increased lipidated LC3, p62, and Cathepsin D levels, in line with decreased HOPS function. We also detected the possible formation of a human VPS39-VPS8 containing hybrid complex.

## Results

### Overexpression of CORVET-specific subunits decreased the amount of HOPS

Human HOPS and CORVET are heterohexamers similarly to the yeast complexes, so the effect of complex-specific subunit levels can be examined in cultured human cells.

**Figure 1:**
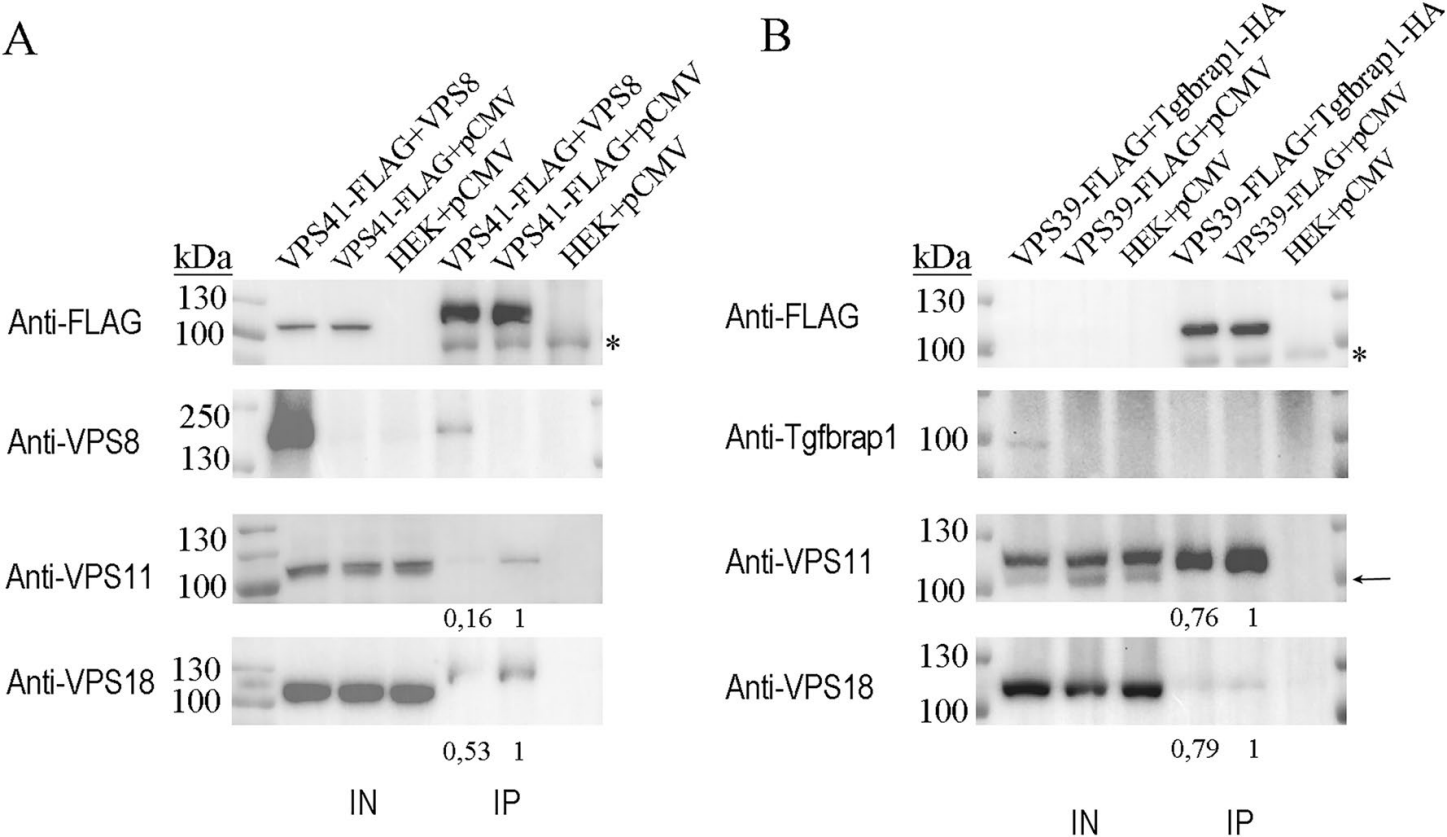
Overexpression of Tgfbrap1 or VPS8 decreases HOPS assembly. HEK293 cells were stably transfected with VPS41-FLAG (**A**) or VPS39-FLAG (**B**) and transiently transfected with VPS8 (**A**) or Tgfbrap1 (**B**), respectively. Total cell lysates were applied on anti-FLAG resin and bound proteins were detected by western blot. Overexpression of CORVET-specific subunits decreases the amounts of VPS41- (**A**) or VPS39-bound (**B**) core subunits (VPS11 and VPS18) compared to control cells (HEK + pCMV). Relative amounts of VPS41- (**A**) or VPS39-bound (**B**) core subunits were calculated by densitometry using Image J and are shown below each panel. Representative blots shown of three experiments, based on which the mean ± error values are: Vps41-FLAG + Vps8_Vps11 blot: 0.31 ± 0.17; Vps41-FLAG + Vps8_Vps18 blot: 0.69 ± 0.35; Vps39-FLAG + Tgf-HA_Vps11 blot: 0.66 ± 0.14; Vps39-FLAG + Tgf-HA_Vps18 blot: 0.63 ± 0.27. Arrows indicate isoform 2 of VPS11 in the INPUT samples. *: unspecific bands.

First, we investigated the effect of CORVET-specific subunits on the intracellular level of HOPS complex. Either Tgfbrap1 or VPS8 were overexpressed in HEK293 cells stably transfected with VPS39-FLAG or VPS41-FLAG, respectively, and after anti-FLAG IP, the amount of core subunits (VPS11 and VPS18) were analysed. VPS41 could precipitate 61% less VPS11 and 47% less VPS18 upon VPS8 overproduction compared to control cells (Fig. 1A). Similarly, VPS39 could bind lower amount of VPS11 (decreased by 24%) and VPS18 (decreased by 21%) when Tgfbrap1 was overexpressed (Fig. 1B). These data suggest that in excess amount, CORVET-specific subunits successfully compete with their HOPS-counterparts, decreasing the amount of assembled HOPS.

Based on the results above, we examined whether these competitions between the HOPS/CORVET counterparts (VPS41/VPS8 and VPS39/Tgfbrap1) are supported by structural data as well. The binding sites of VPS39 and Tgfbrap1 on VPS11 were identified by van der Kant et al.^19^: both proteins bind to the same region of VPS11 (amino acids 774-940), which allows competitive binding. Since the VPS8 binding site of VPS18 was not known, we mapped it with yeast two-hybrid system using truncated VPS18 and full-length VPS8 proteins (additionally we mapped the VPS41 binding site of VPS18 as well). Amino acids 482-854 of VPS18 bind both VPS8 and VPS41, and VPS8 also showed binding to the C-terminal part of VPS18 (aa 855-973) (Fig. 3). Hence, the overlapping binding regions of VPS8 and VPS41 on VPS18 support our IP results and the competitive relationship between the corresponding HOPS/CORVET subunits.

### Overexpression of HOPS-specific subunits did not affect the amount of CORVET

**Figure 2:**
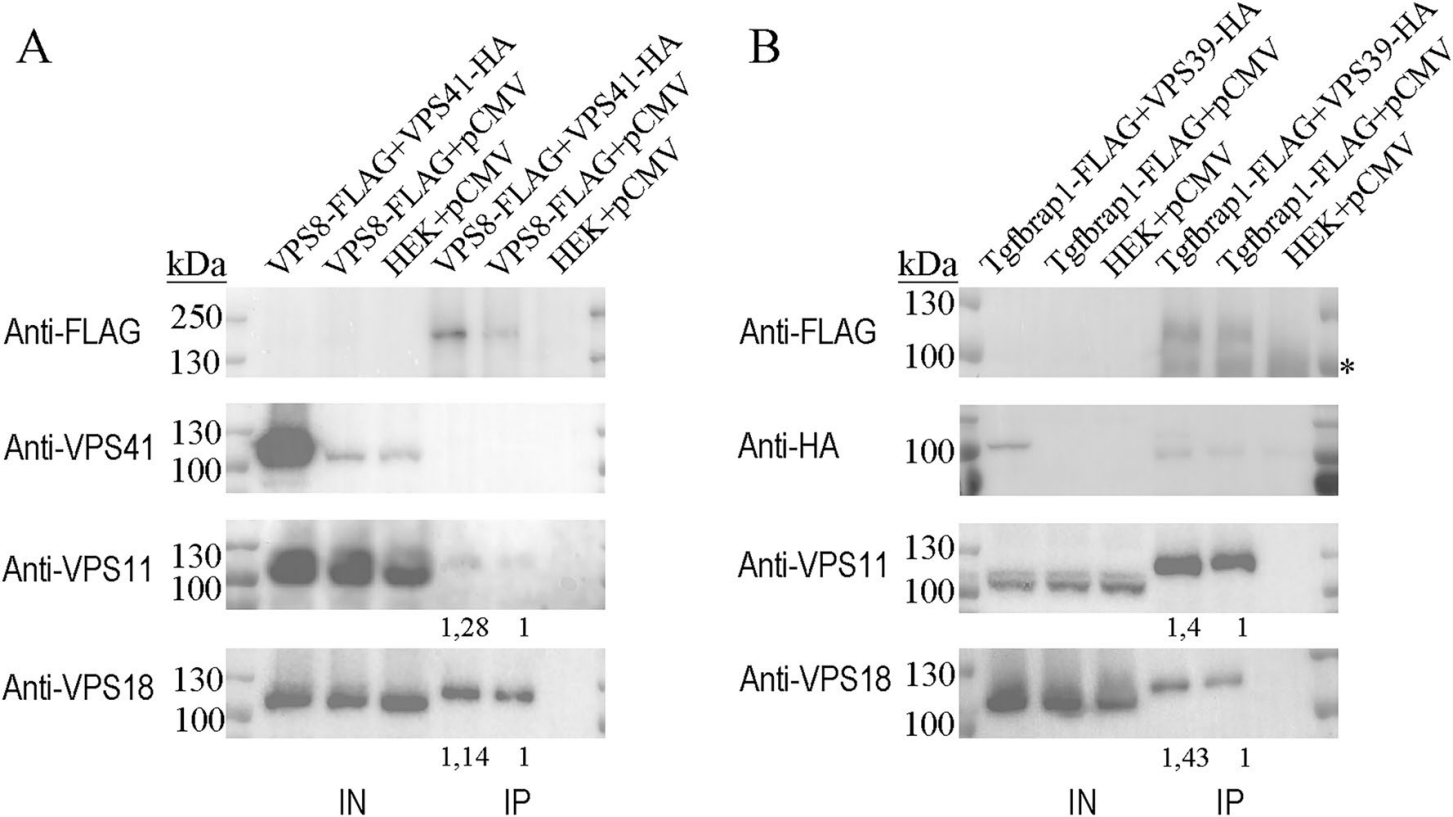
The overproduction of Vps39 or Vps41 does not decrease the amount of CORVET-bound Vps11 and Vps18. HEK293 cells were stably transfected with Vps8-FLAG (**A**) or Tgfbrap1-FLAG (**B**) and transiently transfected with Vps41-HA (**A**) or Vps39-HA (**B**), respectively. Total cell lysates were applied on anti-FLAG resin and bound proteins were detected by western blot. Overexpression of HOPS-specific subunits did not change the amounts of Vps8- (**A**) or Tgfbrap1-bound (**B**) core subunits (Vps11 and Vps18) compared to control cells (HEK + pCMV). Relative amounts of Vps8- (**A**) or Tgfbrap1-bound (**B**) core subunits were calculated by densitometry using Image J and are shown below each panel. Representative blots shown of three experiments, based on which the mean ± error values are: Tgfbrap1-FLAG + Vps39-HA_Vps11 blot: 1.05 ± 0.43; Tgfbrap1-FLAG + Vps39-HA_Vps18 blot: 1.31 ± 0.71; Vps8-FLAG + Vps41-HA_Vps11 blot: 1.52 ± 0.82; Vps8-FLAG + Vps41-HA_Vps18 blot: 1.21 ± 0.25. *: unspecific bands.

Next, we determined the effect of HOPS-specific subunit overexpressions on CORVET. We followed the same logic as above and overexpressed either VPS39 or VPS41 in cells stably transfected with Tgfbrap1-FLAG or VPS8-FLAG, respectively. After anti-FLAG IP, we found no change in the amount of bound core subunits, VPS11 and VPS18, despite the increased levels of VPS39 or VPS41 (Fig. 2). These indicated that elevated amounts of HOPS-specific subunits could not outcompete CORVET-specific Tgfbrap1 and VPS8, and hence the level of assembled CORVET is not affected.

### Overexpression of CORVET-specific subunits inhibited autophagic degradation and caused lysosomal hydrolase accumulation

During macroautophagy, autophagosomes transport their cargo to the lysosomal compartment by fusion with lysosomes. HOPS mediates autophagosome-lysosome fusion^7–9^, besides the fusions of late endosomes. To assess the effect of CORVET-specific subunit overexpression on HOPS function, we examined later steps of the autophagic processes using different methods. Accumulations of the lipidated form of microtubule-associated protein 1A/1B light chain 3B (LC3) and the autophagic cargo p62 are observed if there is a failure of autophagosome-lysosome fusion and can serve as a readout of HOPS function^20^.

To examine the effect of the overexpressed CORVET-specific subunits on HOPS function, we used our HEK293 cell lines stably transfected with FLAG-tagged versions of each subunit. LC3 levels were analysed by western blot (WB). Cells overexpressing CORVET-specific Tgfbrap1 or VPS8 had significantly more lipidated LC3 (LC3-II), similarly to cells lacking the core subunit VPS18 (VPS18KO; both HOPS and CORVET functions are impaired in these cells). HOPS-specific subunits VPS39 or VPS41 did not have the same effect (Fig. 4A and B). Additionally, the autophagic cargo p62 accumulated in cells overproducing VPS8 or Tgfbrap1, just like in VPS18KO cells based on immunocytochemistry (Fig. 4C and D). Again, the surplus of HOPS-specific subunits VPS39 or VPS41 did not elevate the amount of p62 (Fig. 4C and D).

Besides autophagy, HOPS takes part in several other pathways, such as the delivery of lysosomal enzymes from the Golgi to lysosomes. In the absence of VPS11, VPS18, VPS39, or VPS41, the “Golgi-to-lysosome” transport of the aspartic protease Cathepsin D (CathD) enzyme is disturbed in HeLa cells, leading to its accumulation^21^. We applied immunocytochemistry to investigate if the overexpression of VPS8 or Tgfbrap1 affects endogenous CathD levels. Similarly to Vps18KO cells, CathD indeed accumulated in VPS8 and Tgfbrap1 overexpressing cells as well (Fig. 4E and F).

The elevated levels of LC3-II, p62, and CathD in cells with surplus of VPS8 or Tgfbrap1 are indicating decreased HOPS function.

### Hybrid complex containing HOPS-specific VPS39 and CORVET-specific VPS8 can form in HEK293 cells

We sought to investigate the possibility of hybrid complex formation in HEK293 cells. To this end, we overexpressed VPS39-FLAG and untagged VPS8, or VPS39-FLAG and VPS41-HA (control for HOPS). In the anti-FLAG IPs, we could detect VPS8 (indicating the presence of a VPS39–VPS8 hybrid) or VPS41-HA (HOPS) in the eluates (Fig. 5A). Next, we overexpressed Tgfbrap1-FLAG together with VPS41-HA or with untagged VPS8 (control for CORVET). In these experiments, we could detect only VPS8 in the anti-FLAG IP eluate (Fig. 5B). These data indicate that a hybrid complex containing VPS39 and VPS8 can form in HEK293 cells, while we saw no evidence for the formation of VPS41–Tgfbrap1 hybrid complex.

## Discussion

**Figure 3:**
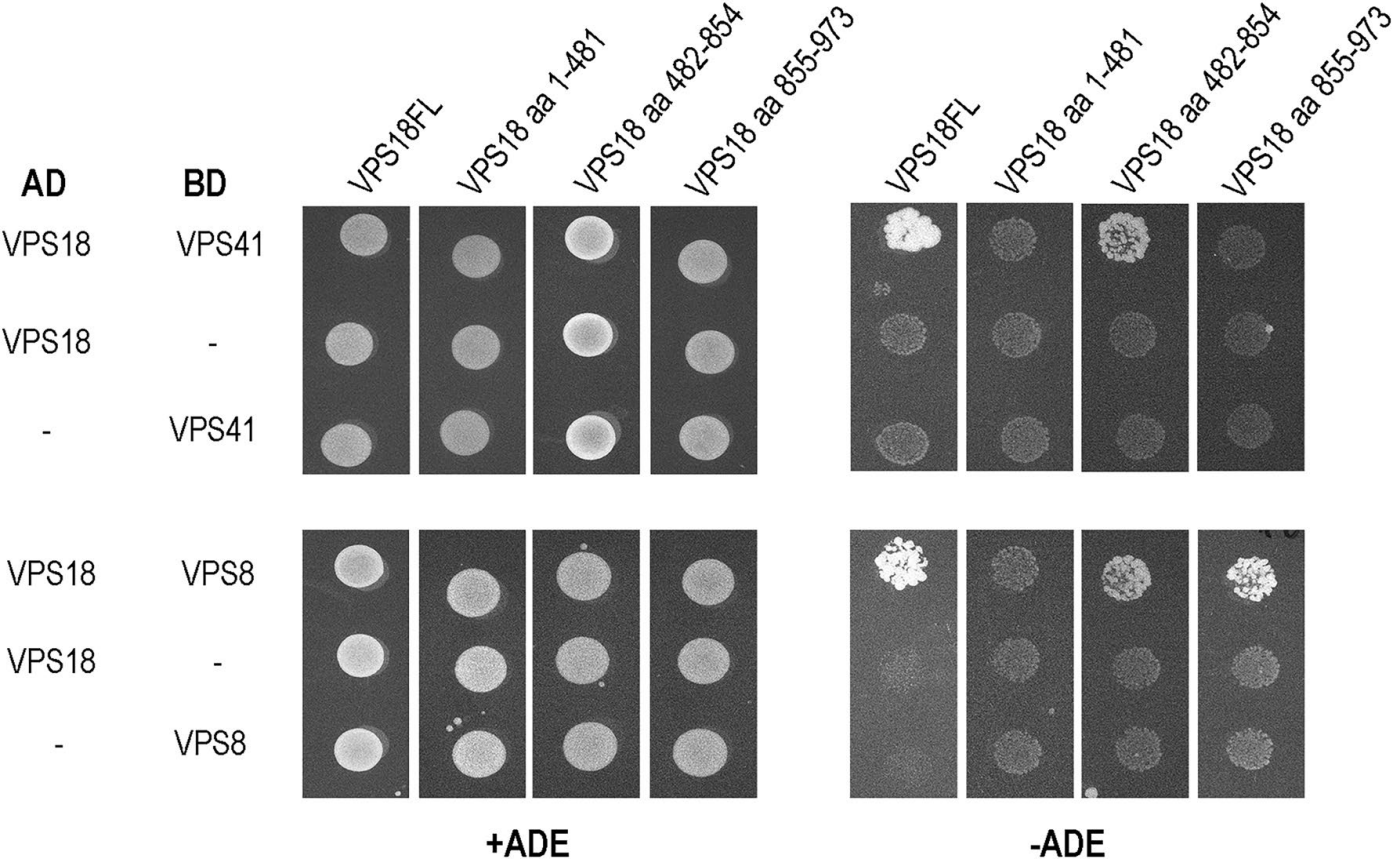
The binding sites of VPS41 and VPS8 on VPS18 overlap. Yeast cells were transformed with pGAD (AD) and pGBKT7 (BD) plasmids containing the coding regions of the indicated full length (VPS18FL, VPS41 and VPS8) or truncated (VPS18 aa) proteins. Cells were then selected on selective media containing (+ ADE) or lacking adenine (−ADE). Full-length proteins VPS18 + VPS41 and VPS18 + VPS8 interact with each other (yeast cells could grow on medium lacking adenine), and both VPS41 and VPS8 bind to truncated VPS18 containing aa 482-854. VPS8 bind to the C-terminus of VPS18 (aa 855-973) as well.

In our study, we investigated the assembly of two endosomal tethering complexes, namely CORVET and HOPS, which act in different stages of endolysosomal trafficking. The existence of subunits shared between the two complexes suggests that cells must keep a fine balance between the levels of HOPS and CORVET and hence changes in the expression level of specific subunits may affect complex assembly. Of note, in yeast, CORVET-specific VPS3 overexpression caused vacuole fragmentation, resembling to a HOPS loss of function phenotype, and the level of HOPS also decreased^5^. Additionally, yeast VPS39 and VPS3 occupy overlapping binding sites on VPS11^17^ and human VPS39 and Tgfbrap1 share their binding sites on VPS11 as well^19^. According to our yeast two-hybrid results, the binding sites of human VPS8 and VPS41 also overlap on VPS18 (aa 482-854) (Fig. 3). These altogether suggest competition between specific subunits and are in line with our overexpression IP results, as elevated level of VPS8/Tgfbrap1 decreased the amount of core subunits VPS11 and VPS18 that are bound with VPS41/VPS39, respectively (Fig. 1).

In yeast, overproduction of VPS8 did not affect HOPS function and the level of VPS41-bound VPS33 (used as readout for assembled HOPS) was unaffected as well^5^. In contrast, overexpression of *Drosophila* VPS8, the only (mini)CORVET-specific subunit, inhibited all HOPS-dependent trafficking routes by outcompeting VPS41 from HOPS and decreased the level of VPS41-bound VPS16, VPS18 and VPS33^18^. Because a smaller version of CORVET exists in flies lacking a VPS3 homolog^14^, in this model only one of the CORVET-specific subunits could be examined. In human, both complexes are hexamers. Our results revealed that both CORVET-specific subunits compete with their HOPS-specific counterparts and as a consequence of their overproduction, less assembled HOPS could be detected (Fig. 1A and B). In contrast, surplus of HOPS-specific subunits VPS39 or VPS41 did not impair CORVET assembly (Fig. 2), similar to previous functional analysis from Drosophila^18^.

**Figure 4:**
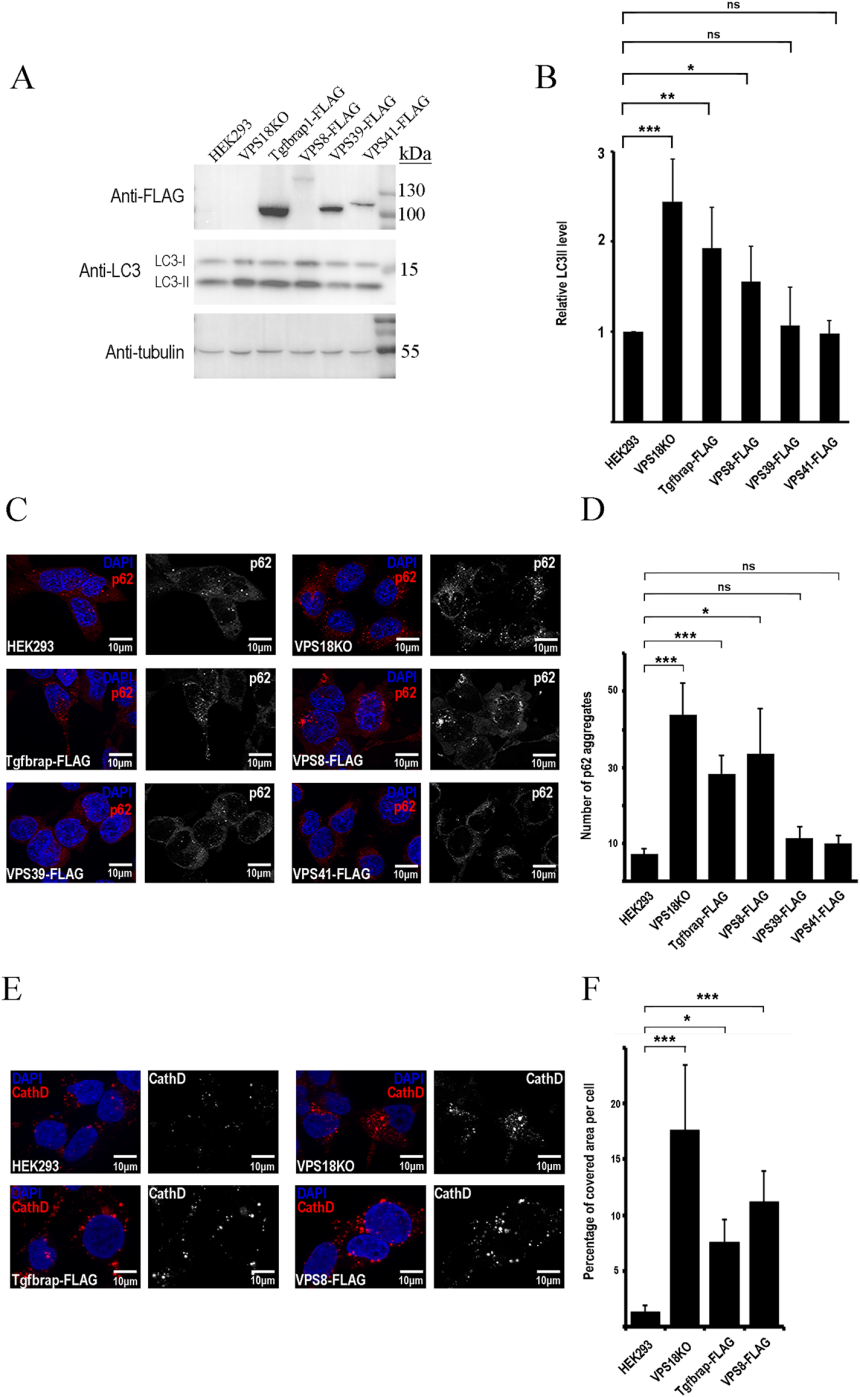
Overexpression of Tgfbrap1 or VPS8 decreases HOPS function. Total cell lysates of VPS18 KO and HEK293 overexpressing different FLAG-tagged HOPS- or CORVET subunits were investigated by western blot. The levels of the overexpressed proteins (anti-FLAG), LC3 and tubulin (loading control) were detected (**A**). Tgfbrap1 and VPS8 overexpression elevated the level of lipidated, autophagosome-associated LC3 (LC3II). The quantification of LC3 blots were done by densitometry using Image J, based on three experiments (**B**). Stably transfected cells were cultured in chambers covered with fibronectin for 24 h, then they were incubated in starvation (amino acid-free) medium for 3 h (**C**) or without starvation (**E**) and after fixation they were incubated with anti-p62 (**C**) or anti-CathD antibody (**E**). Cells overexpressing Tgfbrap1 or VPS8 showed significantly elevated numbers of p62 dots (**D**) and higher percentage of the cell areas were covered by CathD-positive dots (**F**) compared to wild type HEK293 cells. Representative images of three experiments. *, p < 0.05; **, p < 0.01; ***, < 0.001; ns = not significant. In the case of LC3 and p62, where the distributions of the datasets are normal, we applied one-way ANOVA and Tukey or Dunnett post hoc tests. In the case of CathD, we implemented Kruskal–Wallis test because the distributions of the datasets are not normal.

Our data suggest that CORVET-specific subunits may outcompete HOPS-specific ones, and hence they can affect HOPS function. Since HOPS mediates autophagosome-lysosome fusion (besides homotypic fusion of Rab7-positive structures), we determined the levels of lipidated LC3 and the autophagic cargo p62. These proteins accumulate on the autophagosomal membrane and within autophagosomes, respectively, if the fusion process is inhibited. The elevated levels of these proteins in cells overexpressing Tgfbrap1 or VPS8 (CORVET-specific subunits) (Fig. 4) are in line with loss of HOPS function. Similarly, accumulation of Ref(2)P/p62 and lipidated Atg8a is observed in *Drosophila* systematically overexpressing VPS8^18^, while yeast cells with surplus of VPS3 showed fragmented vacuoles, resembling to cells lacking VPS39^5^.

Delivery of proteases from the Golgi to the lysosomes were proven to be HOPS-dependent, as the inactive pro-form of CathD accumulates in HOPS-mutant HeLa cells in so called “HOPS-bodies”^21^. This is in line with the observation that the maturation of CathD is impaired in the brain of VPS18 KO mice^22^ leading to accumulation of the immature form. Our IC results also showed elevated level of CathD in VPS18KO cells, similarly to cells overexpressing VPS8 or Tgfbrap1 (Fig. 4E and F). These phenotypes are similar to VPS11 KO or VPS18 KO HeLa cells, which harbor HOPS-bodies that contain accumulated cathepsins and endocytosed cargo^21^. Taken together, overexpression of human VPS8 and Tgfbrap1 inhibit HOPS function, presumably by displacing their HOPS counterparts.

**Figure 5:**
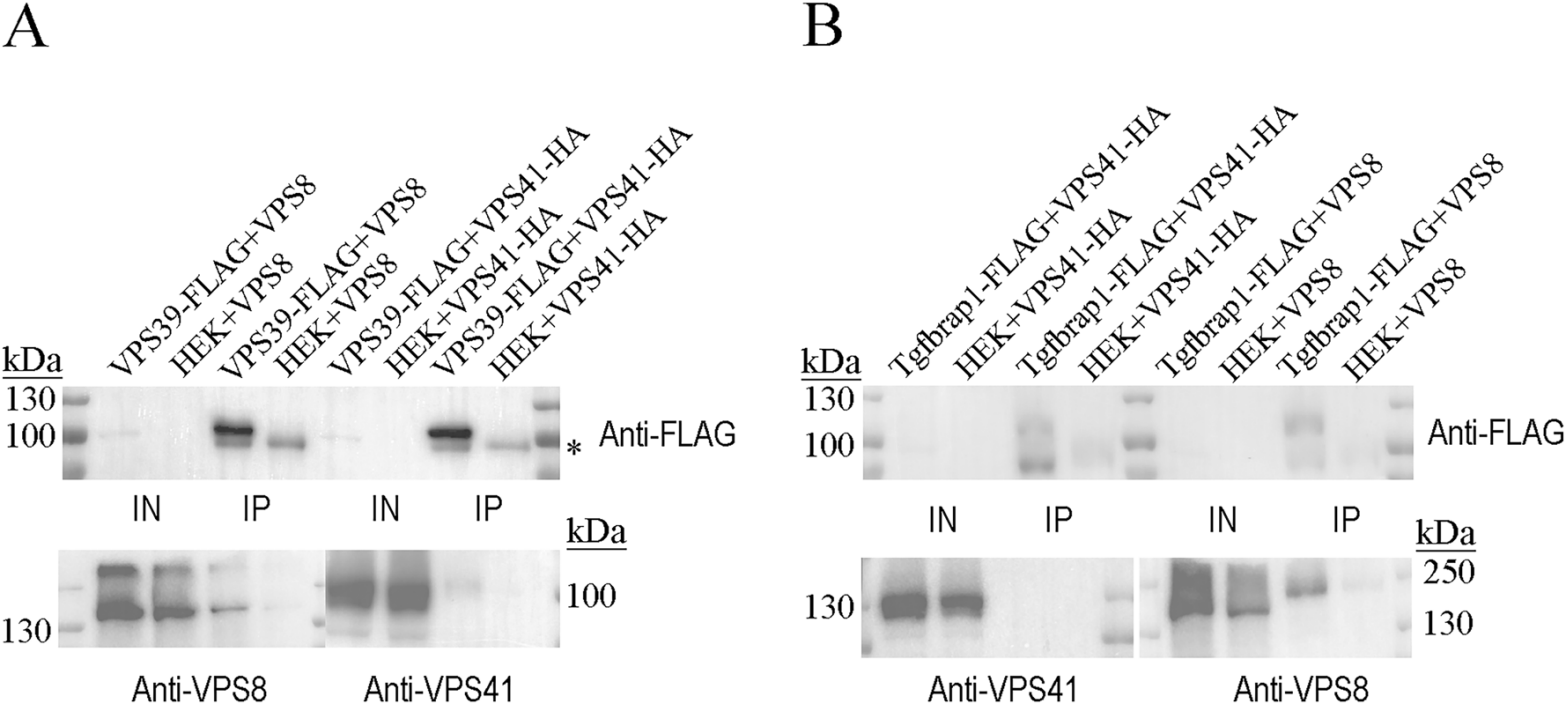
A hybrid complex containing VPS39 and VPS8 can form in HEK293 cells. Cells were transfected with the combination of VPS39-FLAG and VPS8 or with VPS39-FLAG and VPS41-HA. After anti-FLAG IP not just VPS41 (HOPS), but VPS8 (VPS39–VPS8 hybrid) could be detected in the eluates. *: unspecific bands.

Both human complexes are hexamers and they share four core subunits. Thus, there is a chance that CORVET-HOPS hybrid complexes form, just like in yeast cells. According to our data, human VPS39-FLAG could precipitate not only the HOPS-specific VPS41, but also the CORVET-specific VPS8. Thus, a hybrid complex containing VPS39 and VPS8 may form in human cells (Fig. 5A). In contrast, Tgfbrap1-FLAG could not co-precipitate HOPS-specific VPS41, only the CORVET subunit VPS8 (Fig. 5B). Earlier data point to the existence of such hybrid complex in yeast as well, since Vam6/VPS39 can bring down VPS8 (besides other HOPS subunits), however, the amount of VPS8 was much lower compared to its HOPS counterpart VPS41^5^. Additionally, in another study, only a pentamer complex could be identified from cells lacking VPS3, which contained only VPS8 and core subunits without VPS39. In contrast, VPS41 replaces VPS8 in VPS8 mutant cells, and a hexamer VPS41-VPS3 hybrid complex forms^17^. The formation of both hybrid complexes is thus possible in yeast, but the stability and functionality of these are different: the level of the VPS41–VPS3 hybrid complex is likely higher. The only possible hybrid complex (VPS8–VPS39) could not be identified in flies^14^. Recently, hybrid complexes containing class C (core) subunits and VPS41 + VPS3 (Hybrid A) or VPS8 + VPS39 (Hybrid B) were identified in HeLa cells; however, the amount of Hybrid B seemed to be lower than Hybrid A^23^. We have also detected formation of Hybrid B, but not hybrid A. This hybrid complex could as well be an intermediate during CORVET-to-HOPS transition, but our previous Drosophila data indicated that HOPS and CORVET assemble independent of each other^18^. Because Hybrid B was found to play a role in vesicle trafficking after pinocytosis in HeLa cells ^23^, this raises the possibility that it assembles and functions independent of canonical CORVET and HOPS.

Taken together, we show that the amount of CORVET-specific subunits shifts the balance between these two endosomal tethering factors towards CORVET at the expense of assembled HOPS. As expected, this results in impaired HOPS function based on a block of autophagic degradation. Thus, the level of CORVET-specific subunits likely has a strong impact on HOPS function. Our previous analysis in Drosophila supports a model that the CORVET-specific subunit VPS8 is only expressed at detectable levels in highly endocytic cells that have larger-than-usual endosomes (macrophages and nephrocytes, respectively), and endosome progression is only affected in these cell types upon loss of VPS8 function^14^. Bigger-than-usual endosomes in such cells could arise not only from enhanced fusion of early endosomes by CORVET: perhaps somewhat decreased HOPS function may also contribute to vesicle enlargement. We show here that human CORVET-specific subunits have a similar impact on HOPS assembly and function. It will be exciting to see whether CORVET-specific subunit expression also correlates with endocytic activity and endosome size in various mammalian tissues, and whether their loss leads to defects only in highly endocytic cells in knockout mice.

## Materials and methods

### Cloning

To obtain DNA constructs for stably transfected cell lines generation, our previous plasmid constructs were used as a template for amplification of the DNA regions encoding full-length proteins fused with FLAG- or HA-tags^23^. The amplified DNA parts were cloned into mammalian pCMV3 expression vector (SinoBiologicals) to Acc65I—NotI restriction sites using NEBuilder HiFi DNA Assembly Cloning Kit. N-terminal tags were used in the case of VPS41 and VPS39, while C-terminal for VPS8 and Tgfbrap1. We used VPS8 without tag as well, where it is indicated. Plasmids for yeast two-hybrid experiments were established by subcloning DNA parts encoding different VPS18 domains from pGBKT7 vector^23^ into pGADT7 vector, to EcoRI-XhoI restriction sites. The sequences were checked with Sanger sequencing (Microsynth AG, Switzerland).

### Cell culture and transfection

HEK293 (human embryonic kidney) cells were maintained as earlier^24^. Briefly, high glucose Dulbecco’s Modified Eagle Medium (DMEM, Lonza) supplemented with 10% (v/v) heat-inactivated foetal calf serum (FBS, Lonza), 2 mM l-glutamine (Lonza), 100 U/ml penicillin and 100 mg/ml streptomycin were used. Cells were grown in standard conditions (at 37 °C in a humidified atmosphere with 5% CO2). For western blot and IP experiments, 1.5 million HEK293 cells were plated into T25 flasks and transfected after 24 h. Transfection was performed using TransIT-LT-1 (Mirus) transfection reagent and 2500 ng plasmid/flask, according to the manufacturer’s instructions.

For the generation of stably transfected cell lines, cells were selected with Hygromycin (in 200 μg/ml final concentration) for at least 2 weeks (Supplementary Figures).

### Immunoprecipitation

IPs were performed according to our earlier studies^24^, with the following modifications. 24 h after transfection cells were scraped in cold phosphate buffer saline (PBS), then centrifuged and resuspended in lysis buffer (50 mM TRIS–HCl, pH: 7,5, 150 mM NaCl, 1% Triton X-100, 5 mM EDTA, 1 mM PMSF, protease inhibitor cocktail (Roche)), then incubated on ice for 20 min. Samples were centrifuged and 20 μl anti-FLAG beads (Sigma) were added to the lysates. Sample were rotated for 1.5 h at 4 °C. After centrifugation, beads were rotated with wash buffer (same as the lysis buffer without protease inhibitor) for 3 × 10 min at 4 °C. Beads with bound proteins were boiled for 5 min at 100 °C in Laemmli buffer, and loaded onto SDS-PAGE gels for analysis by western blot. A shift of bands in lanes containing immunoprecipitated samples may be observed due to the loading of beads.

### Western blot

Protein samples were made 24 h after transfection. Cells were washed with ice-cold PBS, scraped on ice in lysis buffer (50 mM Tris–HCl pH 8.0, 50 mM KCl, 10 mM EDTA, 1 mM PMSF, 1% Triton X-100) and incubated for 16 min on ice. After centrifugation, the protein amounts of the supernatants were measured by Bradford reagent (Thermo Scientific). Samples were boiled with Laemmli buffer at 100 °C for 5 min. 20 μg protein per sample were applied on the SDS polyacrylamide gel and after electrophoresis, proteins were transferred to PVDF membranes. The following primary antibodies were used: anti-FLAG (Sigma, M2, mouse, 1:2000), anti-HA (Roche, rat, 1:1000), anti-VPS18 (Abcam, rabbit, 1:3000), anti-VPS11 (Sigma, rabbit, 1:500), anti-VPS8 (Atlas, rabbit, 1:500), anti-VPS41 (Abcam, rabbit, 1:1000), anti-Tgfbrap1 (Abcam, rabbit, 1:200), anti-LC3 (Nanotools, mouse, 1:200), anti-p62 (Medical & Biological Laboratories, rabbit, 1:1000), anti-tubulin (DSHB, mouse, 1:800). For development of specific protein bands, horseradish peroxidase (HRP) conjugated secondary antibodies were used in 1:2500 (anti-rabbit, DAKO) or in 1:10.000 (anti-mouse, Sigma) dilution. Protein detections were performed with Immobilon Western Chemiluminescent HRP Substrate (ECL, Millipore). Band intensities were analysed by ImageJ software. Supplementary Figures include unprocessed original version of western blot images.

### Yeast two-hybrid

Yeast strain AH109 was transformed with 300 ng pGADT7 and the same amount of pGBKT7 constructs for each reaction (Frozen EZ Yeast Transformation II, ZYMO Research). The cotransformants were selected on SD (synthetic defined) media, which lacks tryptophan and leucine (SD-WL) and the protein interactions were identified on SD-WL medium without adenine or histidine. The yeasts were incubated for 48 h in 30 °C in every case.

### Immunocytochemistry

Stably transfected cells were plated in eight well chambers covered with fibronectin (50,000 cells/well) and cultured for 24 h. Cells were incubated in starvation medium (containing: 140 mM NaCl, 1 mM CaCl_2_, 1 mM MgCl_2_, 5 mM glucose, 20 mM HEPES) for 3 h then fixed with 4% paraformaldehyde. Rabbit anti-p62 (M&B laboratories) primary and Alexa-Fluor 568 (Thermo Scientific) anti-rabbit antibodies were applied to detect p62-positive structures with Zeiss Axiolmager M2 microscope with ApoTome 2 confocal unit. Images were taken with Zeiss Efficient Navigation (ZEN) software. For revealing CathD, goat anti-CathepsinD (R and D Systems) primary and Alexa-Fluor 568 (Thermo Scientific) anti-goat antibodies were applied.

### Statistics

The datasets were analysed by SPSS (IBM). The distribution’s normality of datasets was checked with Shapiro–Wilk test. In the case of normal distribution one-way ANOVA was implemented. The homogeneity of the variances was checked with the Levene test and if the homogeneity of variances could be assumed, the Tukey post hoc test was applied, but when the homogeneity of variances could not be assumed, we used the Dunnett post hoc test. In the case of non-normal distribution Kruskal–Wallis was implemented.

### Supplementary Information

Supplementary Figures.

## Data Availability

The datasets generated during the current study are available from the corresponding author on reasonable request.

## References

[1] Bonifacino JS, Glick BS (2004). The mechanisms of vesicle budding and fusion. Cell.

[2] Bröcker C (2012). Molecular architecture of the multisubunit homotypic fusion and vacuole protein sorting (HOPS) tethering complex. Proc. Natl. Acad. Sci. U. S. A..

[3] Seals DF, Eitzen G, Margolis N, Wickner WT, Price A (2000). A YptRab effector complex containing the Sec1 homolog Vps33p is required for homotypic vacuole fusion. Proc. Natl. Acad. Sci. U. S. A..

[4] Wurmser AE, Sato TK, Emr SD (2000). New component of the vacuolar class C-Vps complex couples nucleotide exchange on the Ypt7 GTPase to SNARE-dependent docking and fusion. J. Cell Biol..

[5] Peplowska K, Markgraf DF, Ostrowicz CW, Bange G, Ungermann C (2007). The CORVET tethering complex interacts with the yeast Rab5 Homolog Vps21 and is involved in endo-lysosomal biogenesis. Dev. Cell.

[6] Balderhaar HJK (2013). The CORVET complex promotes tethering and fusion of Rab5/Vps21-positive membranes. Proc. Natl. Acad. Sci. U. S. A..

[7] Angers CG, Merz AJ (2009). HOPS interacts with Apl5 at the vacuole membrane and is required for consumption of AP-3 transport vesicles. Mol. Biol. Cell.

[8] Balderhaar HJK, Ungermann C (2013). CORVET and HOPS tethering complexes - coordinators of endosome and lysosome fusion. J. Cell Sci..

[9] Takáts S (2014). Interaction of the HOPS complex with Syntaxin 17 mediates autophagosome clearance in Drosophila. Mol. Biol. Cell.

[10] Lörincz P (2017). Rab2 promotes autophagic and endocytic lysosomal degradation. J. Cell Biol..

[11] Van Der Kant R (2013). Late endosomal transport and tethering are coupled processes controlled by RILP and the cholesterol sensor ORP1L. J. Cell Sci..

[12] McEwan DG (2015). PLEKHM1 regulates autophagosome-lysosome fusion through HOPS complex and LC3/GABARAP proteins. Mol. Cell.

[13] Zhen Y, Stenmark H (2015). Cellular functions of Rab GTPases at a glance. J. Cell Sci..

[14] Lőrincz P (2016). MiniCORVET is a Vps8-containing early endosomal tether in drosophila. Elife.

[15] Lachmann J, Glaubke E, Moore PS, Ungermann C (2014). The Vps39-like TRAP1 is an effector of Rab5 and likely the missing Vps3 subunit of human CORVET. Cell. Logist..

[16] Perini ED, Schaefer R, Stöter M, Kalaidzidis Y, Zerial M (2014). Mammalian CORVET is required for fusion and conversion of distinct early endosome subpopulations. Traffic.

[17] Ostrowicz CW (2010). Defined subunit arrangement and rab interactions are required for functionality of the HOPS tethering complex. Traffic.

[18] Lőrincz P (2019). Vps8 overexpression inhibits HOPS-dependent trafficking routes by outcompeting Vps41/Lt. Elife.

[19] Van Der Kant R (2015). Characterization of the mammalian CORVET and HOPS complexes and their modular restructuring for endosome specificity. J. Biol. Chem..

[20] Jiang P (2014). The HOPS complex mediates autophagosome-lysosome fusion through interaction with syntaxin 17. Mol. Biol. Cell.

[21] van der Beek J, de Heus C, Sanza P, Liv N, Klumperman J (2024). Loss of the HOPS complex disrupts early-to-late endosome transition, impairs endosomal recycling and induces accumulation of amphisomes. Mol. Biol. Cell.

[22] Peng C (2012). Ablation of vacuole protein sorting 18 (Vps18) gene leads to neurodegeneration and impaired neuronal migration by disrupting multiple vesicle transport pathways to lysosomes. J. Biol. Chem..

[23] Terawaki S, Vasilev F, Moriwaki T, Otomo T (2023). HOPS, CORVET and newly-identified Hybrid tethering complexes contribute differentially towards multiple modes of endocytosis. Sci. Rep..

[24] Simon-Vecsei Z (2021). Identification of new interactions between endolysosomal tethering factors. J. Mol. Biol..

