# Supplementary material for: CORVET-specific subunit levels determine the balance between HOPS/CORVET endosomal tethering complexes
Source: Sci Rep. 2024 May 2;14:10146. doi: 10.1038/s41598-024-59775-0 (PMC11066007; doi:10.1038/s41598-024-59775-0)

Full membrane blots

Figure 1/A anti-FLAG

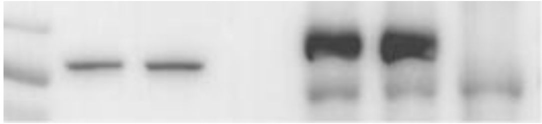

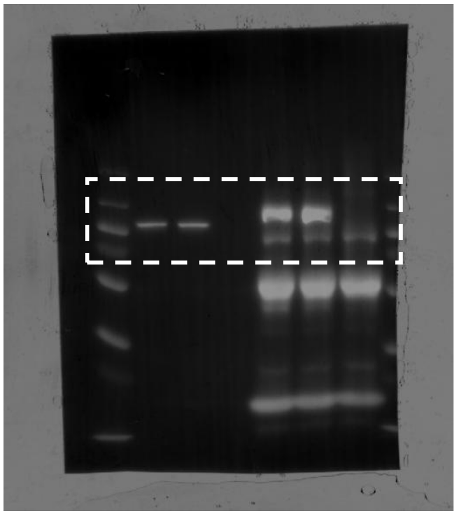

Figure 1/A anti-Vps8

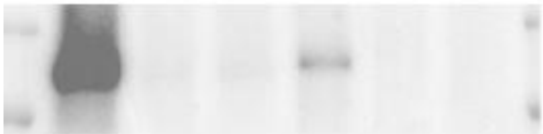

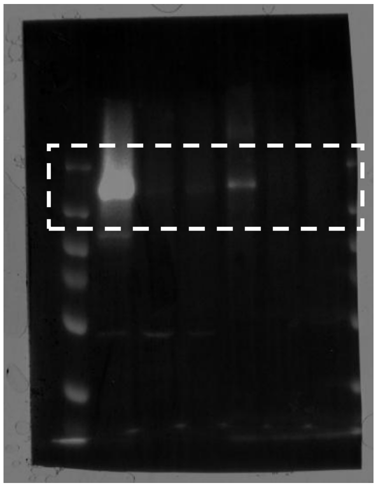

Figure 1/A anti-Vps11

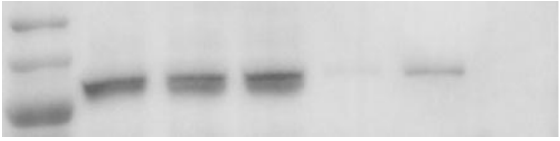

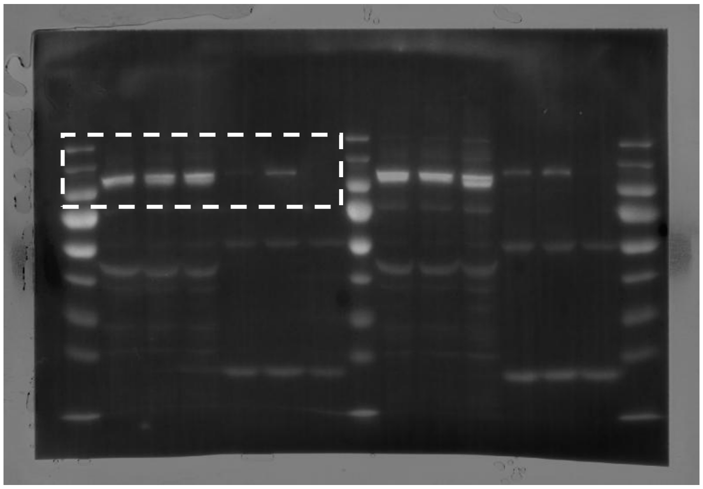

Figure 1/A anti-Vps18

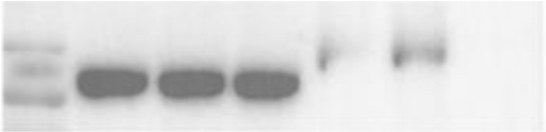

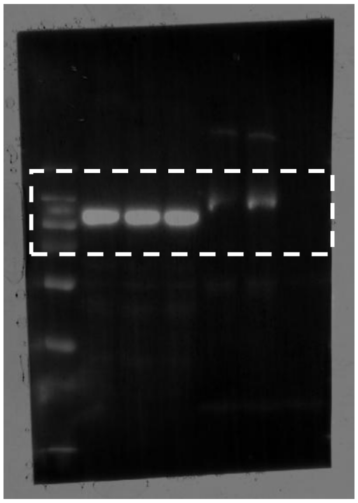

Figure 1/B anti-FLAG

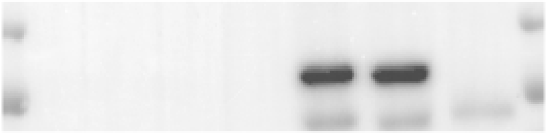

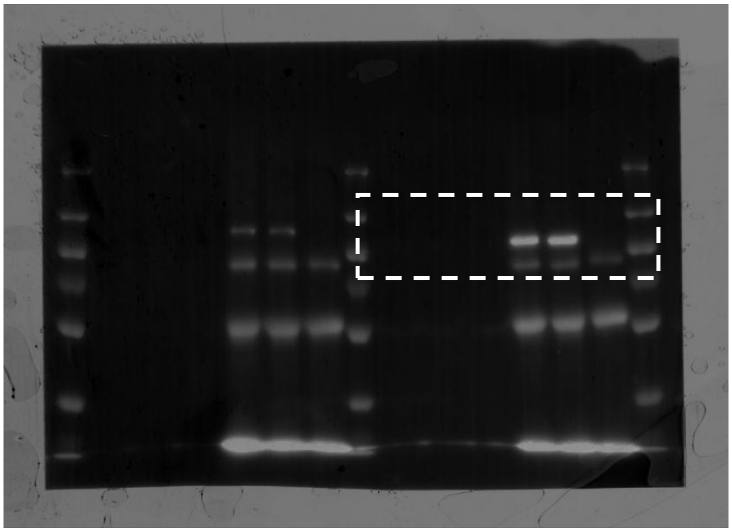

Figure 1/B anti-Tgfbrap1

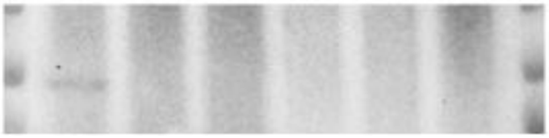

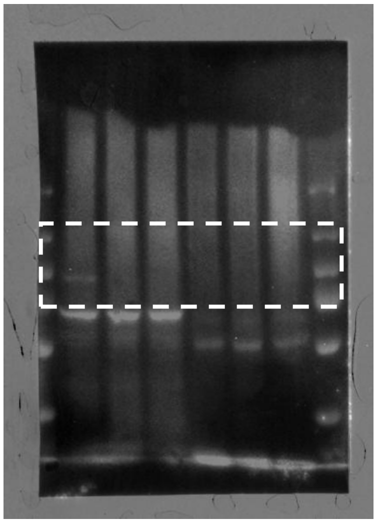

Figure 1/B anti-Vps11

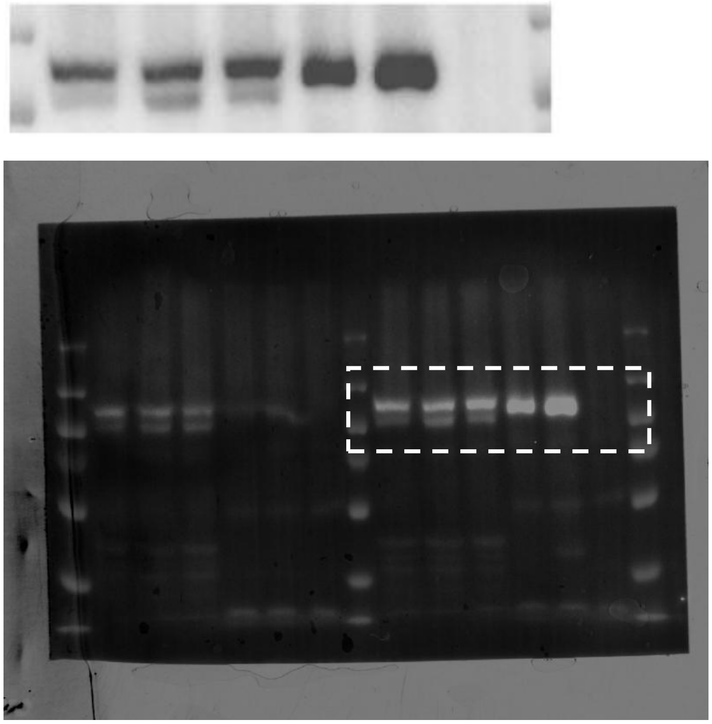

Figure 1/B anti-Vps18

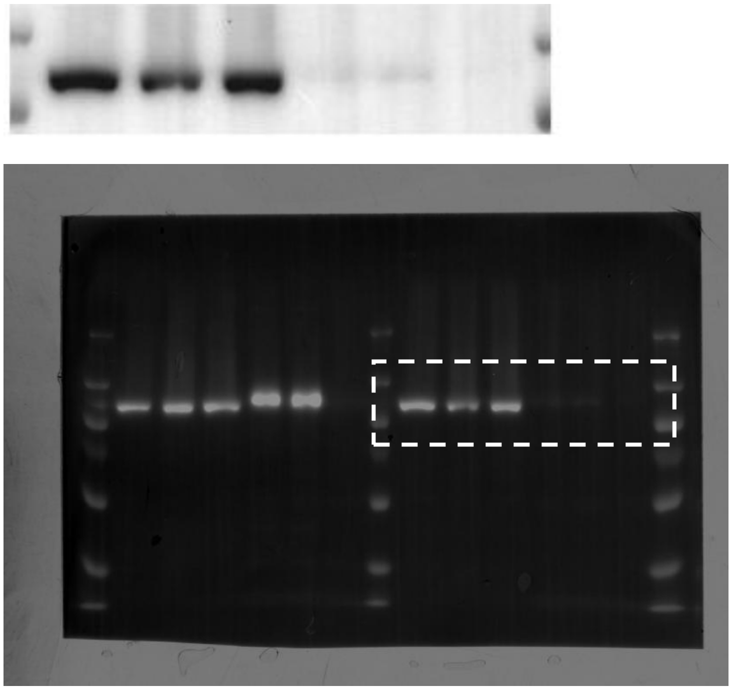

Figure 2A anti-FLAG

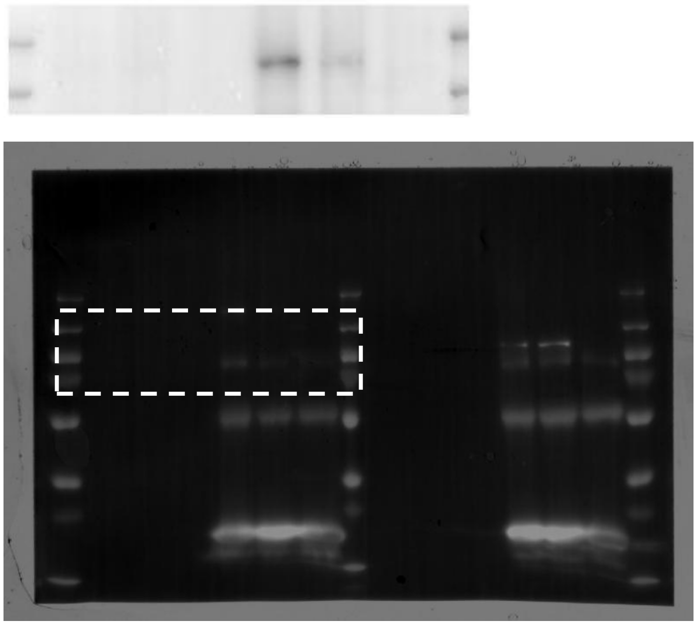

Figure 2A anti-Vps41

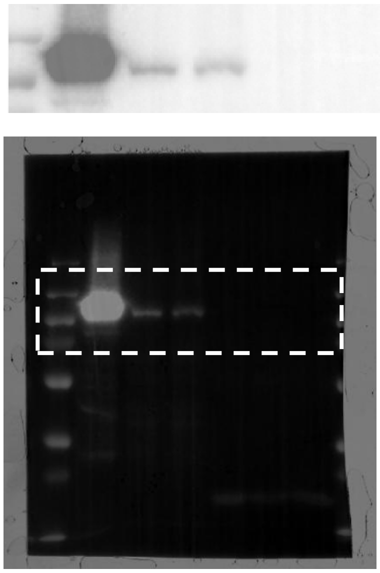

Figure 2A anti-Vps11

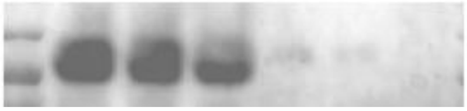

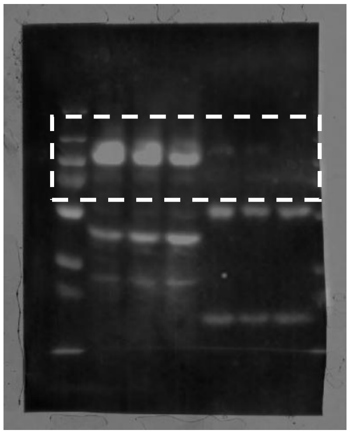

Figure 2A anti-Vps18

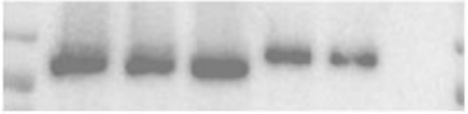

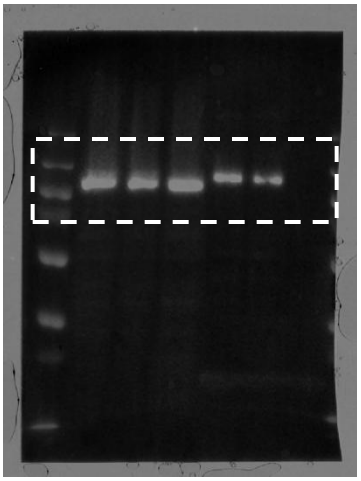

Figure 2B anti-FLAG

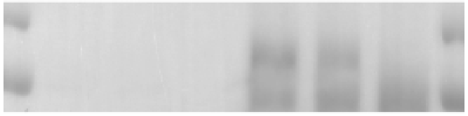

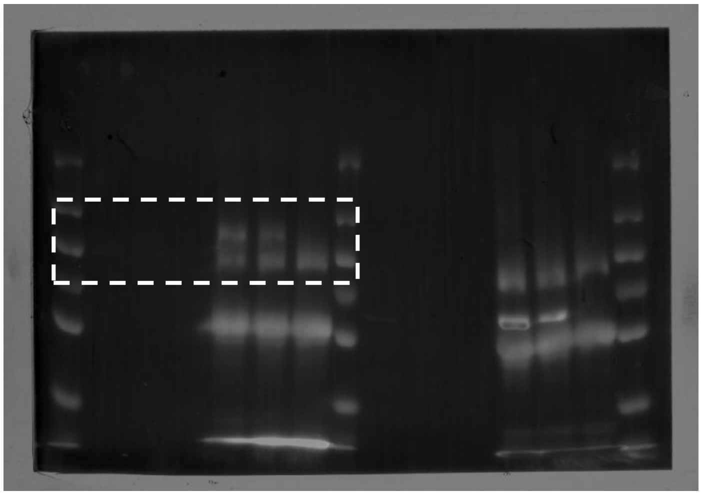

Figure 2B anti-HA

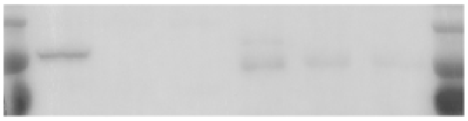

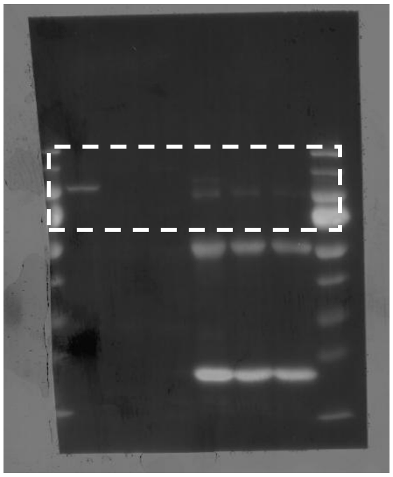

Figure 2B anti-Vps11

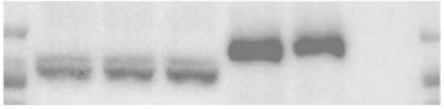

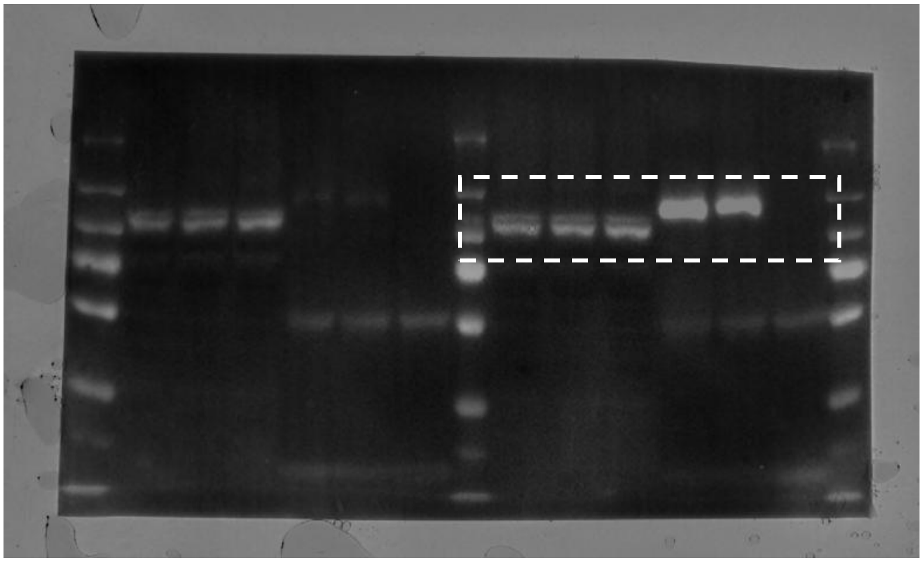

Figure 2B anti-Vps18

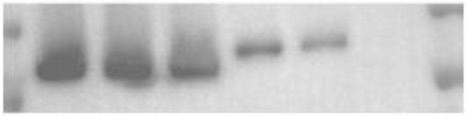

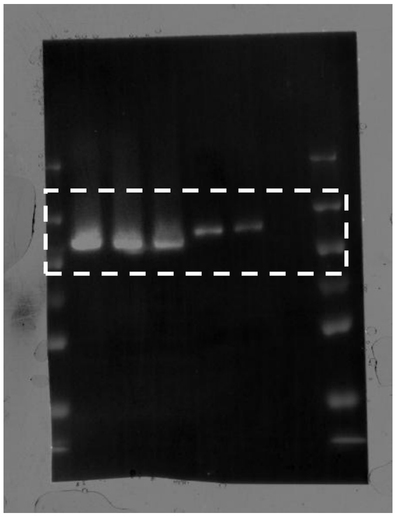

Figure 4/A anti-FLAG

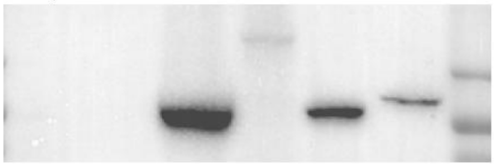

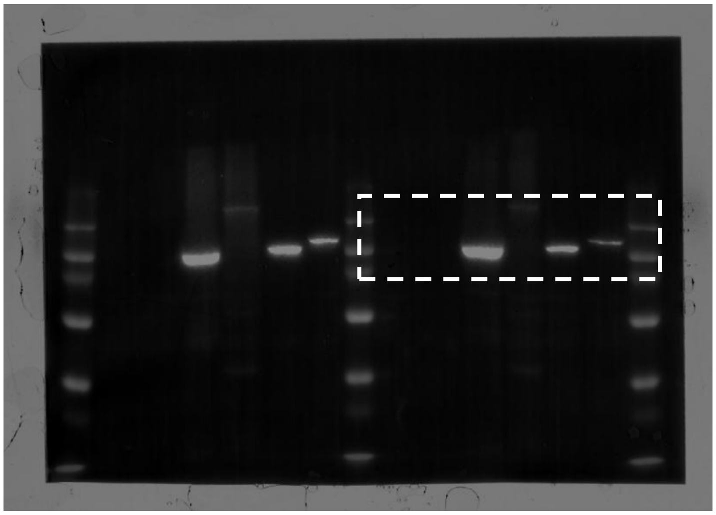

Figure 4/A anti-LC3

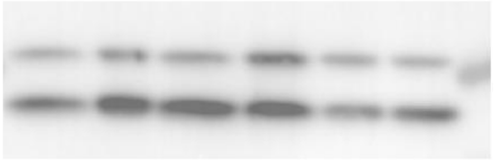

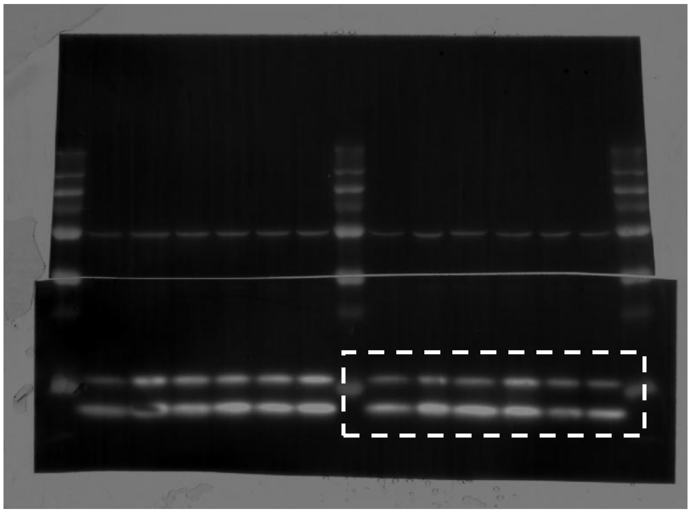

Figure 4/A anti-tubulin

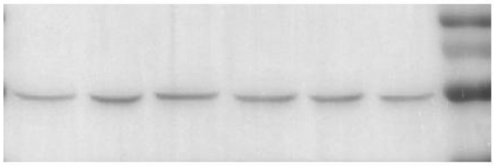

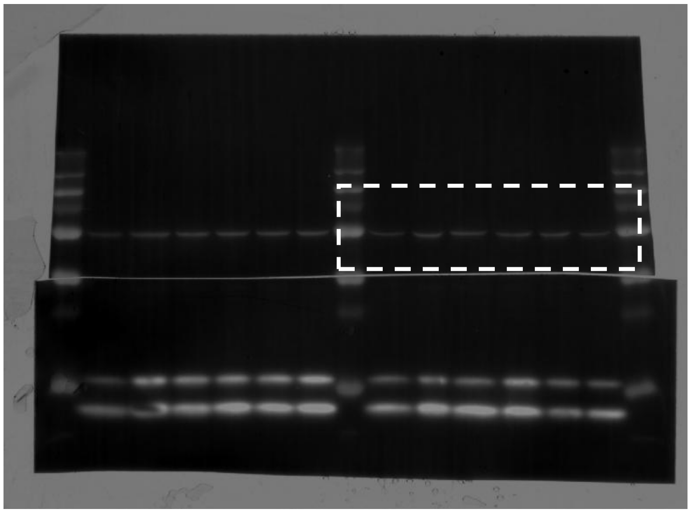

Figure 5A anti-FLAG

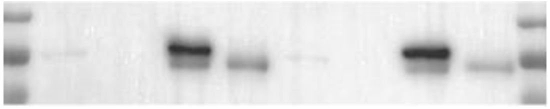

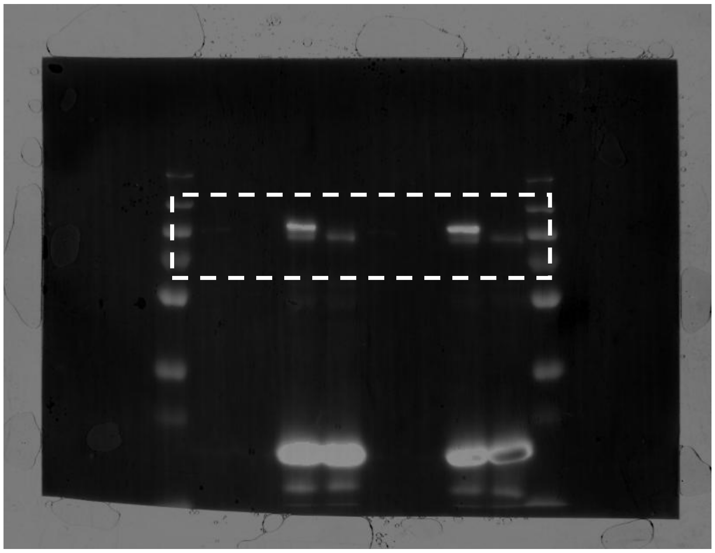

Figure 5A anti-Vps8

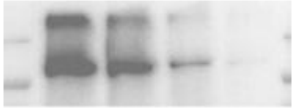

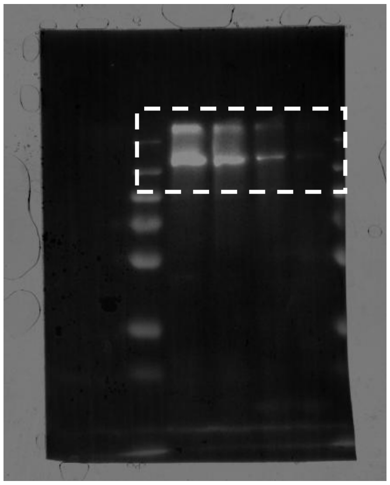

Figure 5A anti-Vps41

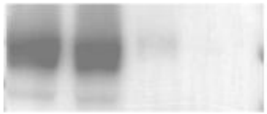

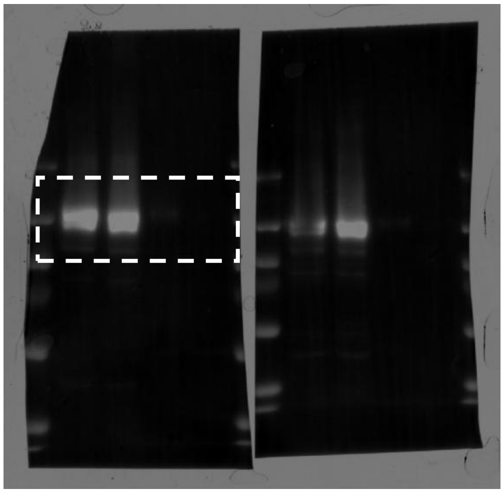

Figure 5B anti-FLAG

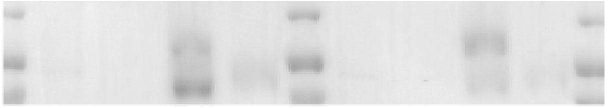

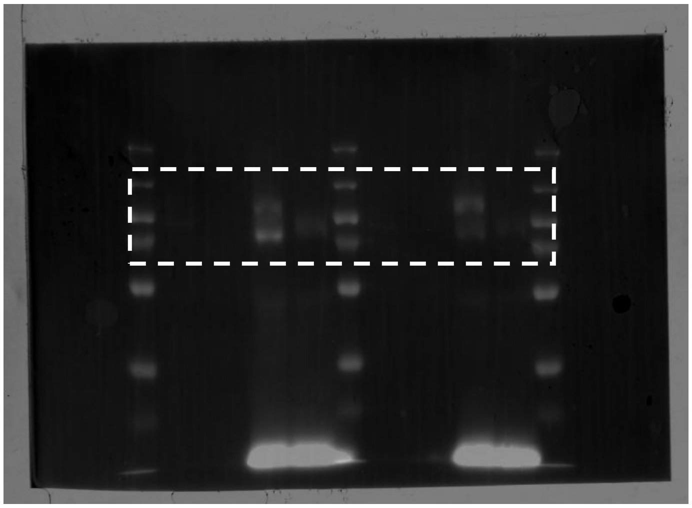

Figure 5B anti-Vps41

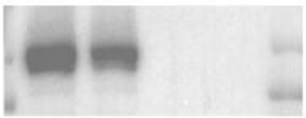

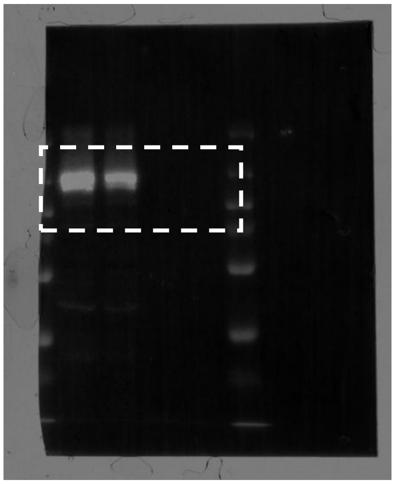

Figure 5B anti-Vps8

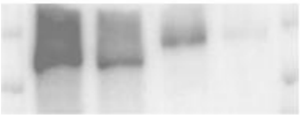

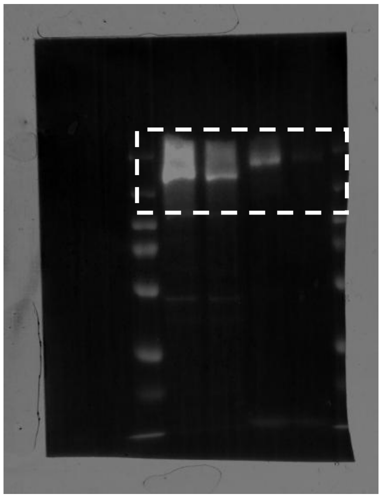

Supplement: Supplementary file 1 — Supplementary Figures. [file 41598_2024_59775_MOESM1_ESM.pdf]
